# Elektronisches Lernen für Studenten in der Hals-Nasen-Ohren-Heilkunde durch Nutzung des Content-Management-Systems ILIAS

**DOI:** 10.1007/s00106-021-01008-1

**Published:** 2021-02-03

**Authors:** Sara M. van Bonn, Jan S. Grajek, Wilma Großmann, Hans E. Bernd, Stefanie Rettschlag, Robert Mlynski, Nora M. Weiss

**Affiliations:** Klinik und Poliklinik für Hals-Nasen-Ohrenheilkunde, Kopf- und Halschirurgie „Otto Körner“, Universitätsmedizin Rostock, Doberaner Str. 137–139, 18057 Rostock, Deutschland

**Keywords:** E‑Learning, Medizinstudierende, COVID-19-Pandemie, Hals-Nasen-Ohren-Heilkunde, Kommunikation, E‑Learning, Medical students, Covid-19 pandemic, Otorhinolaryngology, Communication

## Abstract

**Hintergrund:**

Der Präsenzunterricht ist die vorherrschende Lehrmethode der Universitäten, ist jedoch im Rahmen der digitalen Transformation und des zunehmenden Zugriffs auf Online-Lehrmaterialien zu hinterfragen. Ziel dieser Studie war es zu evaluieren, inwiefern das elektronische Lernen (E-Learning) online als Ersatz für das herkömmliche Anwesenheitspraktikum für Hals-Nasen-Ohren-Heilkunde genutzt werden kann.

**Material und Methoden:**

Ein vollständig digitales elektronisches Lernkonzept wurde auf der Online-Lernplattform ILIAS erstellt und zur Verfügung gestellt. Die teilnehmenden Studierenden wurden in das elektronische Lernprogramm eingewiesen. Es wurden 4 Lerneinheiten (äußerer Hals [I], Rachen/Kehlkopf [II], Nase [III], Ohr [IV]) eingerichtet. Nach jeder Lerneinheit erfolgte abschließend ein themenentsprechender Multiple-Choice-Test. Sowohl vor als auch nach Durchführung der Lernkurse wurden die Studierenden gebeten, an der Evaluation teilzunehmen.

**Ergebnisse:**

Insgesamt 105 Studierende nahmen vor und 85 Studierende nach erfolgtem elektronischem Lernprogramm an der Evaluation teil. Die Mehrheit der Studierenden (52,94 %) gab nach Durchführung der Kurse einen signifikant höheren Zufriedenheitswert bezüglich des Inhalts, der Darstellung der Lernsequenzen und der eigenen Kontrolle über Lerntempo bzw. Zeiteinteilung gegenüber dem Zeitpunkt vor Absolvierung des elektronischen Lernprogramms (34,29 %) an (*p* < 0,0001). Ein Großteil der Studierenden (54,12 %) wünscht sich das elektronische Lernangebot zusätzlich zur Präsenzlehre.

**Schlussfolgerungen:**

Das elektronische Lernprogramm ist ein vielversprechender Ansatz als Alternative bzw. Ergänzung zum traditionellen Lernen bzw. dem Lernen durch die Teilnahme an Präsenzveranstaltungen. Eine Erweiterung der digitalen Lehre kann auf der Basis dieser Untersuchung ausdrücklich unterstützt werden.

## Abstract (en)

**Background:**

With increasing access to online teaching materials, traditional teaching methods at universities need to be questioned in the context of digital transformation. The aim of this study was to evaluate whether electronic learning may serve as or replace conventional internship in otorhinolaryngology.

**Materials and methods:**

A completely digital electronic learning concept was created and made available at the online learning platform ILIAS. Students were introduced to the program. Four learning units (neck [I], pharynx/larynx [II], nose [III], ear [IV]) were set up, with a topic-related multiple-choice test at the end of each unit. The students took part in the evaluation before and after completion of the course.

**Results:**

A total of 105 students participated in the evaluation before and 85 students after the electronic learning program. After completing the courses, the majority of students (52.94%) stated to be more satisfied with the content and the presentation of the learning sequences as well as with their own self-control concerning learning pace and time management compared to the situation before completing the program (34.29%; *p* < 0.0001). The majority of students (54.12%) stated that they would appreciate the electronic learning program in addition to practical internship.

**Conclusion:**

The electronic learning program is a promising approach to supplement traditional learning and internships. An expansion of digital teaching proposals should be supported based on this study.

Elektronisches Lernen (E-Learning) bezeichnet ein umfassendes Konzept für selbstbestimmte und computergestützte Lehr- und Lernprozesse [1, 2]. In elektronischem Format verfügbare Lehrmaterialien verzeichnen in den letzten Jahren eine wachsende Popularität. So rückt auch in der universitären Lehre die Verwendung von elektronischen Lehrmaterialien immer mehr in den Mittelpunkt [3–5]. Sie bietet die Möglichkeit der Nutzung unterschiedlicher Formen der Visualisierung sowie den Vorteil einer individualisierbaren Lernform unabhängig von Ort und Zeit [6]. Zudem kann durch eine umfangreiche Informationsvermittlung auch in einem internationalen Umfeld leicht zugängliches Wissen erworben und interaktiv überprüft werden [7, 8]. Aktuelle Übersichtsarbeiten zeigen, dass digitale Lehrmittel ein nützliches Instrument für medizinische Ausbildungszwecke darstellen und dabei der traditionellen Vorlesung nicht unterlegen sind [9, 10]. Ebenso wird beschrieben, dass Medizinstudenten, welche elektronische Lernprogramme nutzen, subjektiv mit ihrer Lernerfahrung zufriedener sind im Vergleich zu den Studierenden, welche traditionelle Lernmethoden anwenden [6, 11].

Das reguläre Blockpraktikum für Hals-Nasen-Ohren-Heilkunde, welches bei konventionellen Studiengängen im 7. und 8. Semester stattfindet, dient der praktischen Verfestigung der bisher vermittelten Inhalte der Vorlesung. Der Präsenzunterricht ist die vorherrschende Lehrmethode der Universitäten, ist jedoch im Rahmen der digitalen Transformation und des zunehmenden Zugriffs auf Online-Lehrmaterialien zu hinterfragen. Im Rahmen der anhaltenden COVID-19-Pandemie hat das elektronische Lernen durch Einschränkungen der Präsenzlehre zusätzlich an Bedeutung gewonnen. Zusammen mit den Auswirkungen der COVID-19-Pandemie auf die universitäre Krankenversorgung hat sich die Entwicklung von der analogen zur digitalen Lehre beschleunigt [12]. Bei der Erprobung neuer Lehrmaterialien und Methoden ist neben objektiven Verfahren wie Prüfungen auch eine Evaluation des subjektiven Nutzens von Bedeutung. Ziel dieser Studie war es deshalb, elektronisches Lernen zu evaluieren, um Voraussetzungen zu schaffen, es als Ergänzung für herkömmliche Präsenzpraktika zu nutzen.

## Methodik

Während des Sommersemesters 2020 wurde das elektronische Lernprogramm der Klinik und Poliklinik für Hals-Nasen-Ohrenheilkunde der Universitätsmedizin Rostock im Web-Content-Management-System ILIAS aufgebaut.

### Lernplattform ILIAS

Das Open-Source-Produkt ILIAS ist ein kostenloses, von vielen Hochschulen eingesetztes http-Protokoll (ILIAS open source e‑Learning e. V., Köln) für die internetbasierte Bereitstellung von Lehr- und Lernmaterialien. Alle Studierenden, welche für das Blockpraktikum im Sommersemester 2020 eingeteilt waren, wurden über das Projekt informiert und über die Teilnahme an der anschließenden Evaluation aufgeklärt.

### Erstellen der Lehrinhalte

Es wurden 4 Lernkurse zu den übergeordneten Themen: äußerer Hals [I], Rachen/Kehlkopf [II], Nase [III], Ohr [IV] erstellt. Jedes der Module wurde für eine Lernzeit von ca. 60 min konzipiert. Die Online-Kurse beinhalten Text mit Bilddateien bzw. Schemata sowie selbstgedrehte High-Definition(HD)-Lehrvideos. Nach jeder Lerneinheit folgte abschließend ein themenentsprechender Multiple-Choice-Test bestehend aus 10 Fragen, um den Lernerfolg zu überprüfen.

### Livestream-Operationen

Als Zusatzangebot wurden Operationen als Live-Stream in HD-Qualität aus dem Operationssaal für die Übertragung von Operationen zur Verfügung gestellt. Die Aufnahmen von einem volldigitalen 3‑D-Operationsmikroskop (Arriscope Evo2 HNO, Fa. MSI, München, Deutschland), einer Umfeldkamera (EVI-HD7V, Fa. Sony, Tokio, Japan) sowie die Tonspur wurden über eine LAN-Schnittstelle an einen Mediendienstleister (Fa. pripares, München, Deutschland) übertragen. Die Daten wurden vom Dienstleister transformiert und anschließend über einen eigenen Kanal bei YouTube (Fa. Google LLC, Mountain View, CA, USA) mit einer Chatfunktion online gestellt. Studierende konnten so über gängige Endgeräte von jedem Standort in Deutschland sowie weltweit auf den Stream zugreifen, Fragen zu den chirurgischen Schritten stellen und mit dem Chirurgen kommunizieren. Die Teilnahme am Live-Stream war freiwillig.

### Evaluation

Vor Beginn und nach Abschluss der Lernkurse auf der ILIAS-Plattform wurden die Studierenden gebeten, einen Evaluationsbogen auszufüllen (Tab. 1). Die ersten 7 Punkte der Fragebögen waren zu beiden Untersuchungszeitpunkten identisch. Alle Aussagen der Evaluationsfragebögen waren auf 5‑stufigen Likert-Skalen von 1 („trifft vollkommen zu“) bis 5 („trifft nicht zu“) zu beantworten.

**Table 1:** 

	Fragebogen Teil 1	Fragebogen Teil 2
1.	E‑Learning empfinde ich als hilfreich, da es meine eigene Lerngeschwindigkeit berücksichtigt	E‑Learning empfinde ich als hilfreich, da es meine eigene Lerngeschwindigkeit berücksichtigt
2.	E‑Learning würde zu einer höheren Bereitschaft der Studierenden zur Teilnahme an Kursen und Vorlesungen führen	E‑Learning würde zu einer höheren Bereitschaft der Studierenden zur Teilnahme an Kursen und Vorlesungen führen
3.	Das Fach HNO-Heilkunde eignet sich gut für den Einsatz von E‑Learning	Das Fach HNO-Heilkunde eignet sich gut für den Einsatz von E‑Learning
4.	Die studentische Ausbildung in der HNO-Heilkunde setzt einen Arzt-Studierenden-Kontakt voraus	Die studentische Ausbildung in der HNO-Heilkunde setzt einen Arzt-Studierenden-Kontakt voraus
5.	E‑Learning bietet eine gute Alternative zum herkömmlichen HNO-Anwesenheitspraktikum	E‑Learning bietet eine gute Alternative zum herkömmlichen HNO-Anwesenheitspraktikum
6.	E‑Learning in der HNO-Heilkunde sollte zusätzlich zum Anwesenheitspraktikum zur Verfügung gestellt werden	E‑Learning in der HNO-Heilkunde sollte zusätzlich zum Anwesenheitspraktikum zur Verfügung gestellt werden
7.	Ich wünsche mir ein größeres Angebot für E‑Learning-Kurse und Vorlesungen	Ich wünsche mir ein größeres Angebot für E‑Learning-Kurse und Vorlesungen
8.	Die Bereitstellung eines E‑Learning-Programms sorgt für eine standardisierte und gleichbleibende Lehrqualität (unabhängig von Ort, Zeit, Dozent*in)	Durch das E‑Learning haben sich meine praktischen Fähigkeiten verbessert
9.	E‑Learning kann mir helfen, meinen Studienplan flexibler zu gestalten (z. B. Nebenjob, Elternbesuche)	Durch das E‑Learning der HNO ist mein Interesse am Fach gewachsen, und ich bin motivierter, mich intensiver damit zu befassen
10.	Der Begriff „E-Learning“ ist mir geläufig	Das E‑Learning der HNO-Heilkunde ist strukturiert und logisch aufgebaut
11.	Das E‑Learning-Programm der Universitätsmedizin Rostock nutze ich	Ich habe das Gefühl, gut für das Fachgebiet HNO vorbereitet zu sein
12.	Das E‑Learning-Angebot der Universitätsmedizin Rostock ist mir bekannt	Das E‑Learning-System ist benutzerfreundlich aufgebaut
13.	Wie häufig würden Sie ein entsprechendes HNO-Programm nutzen?	Ich konnte mit in kurzer Zeit viel Wissen aneignen
14.	–	Die Qualität der Lerneinheiten war hoch
15.	–	Ich konnte die Lerneinheiten von verschiedenen Standorten aus abrufen
16.	–	Die Verbindung zum Server beim Bearbeiten der Lerneinheiten war stabil
17.	–	E‑Learning ist ein zeitgemäßes Format
18.	–	Wie oft haben Sie das E‑Learning-Programm genutzt?

*HNO* Hals-Nasen-Ohren

### Statistik

Die statistischen Analysen erfolgten mit GraphPad Prism (Version 8, Fa. GraphPad Software, La Jolla, CA, USA). Das Signifikanzniveau wurde auf *p* < 0,05 festgesetzt. Die Normalverteilung wurde grafisch mit Quantil-Quantil-Diagrammen („quantile-quantile plots“) getestet. Wenn nicht anders angegeben, werden die Ergebnisse als Mittelwerte mit Standardabweichung (SD) oder als absolute Werte mit Prozenten angegeben. Der Wilcoxon-Test wurde durchgeführt, um Unterschiede zwischen der Beantwortung des Fragebogens vor und nach Durchführung des elektronischen Lernprogramms zu untersuchen.

## Ergebnisse

**Figure 1:**
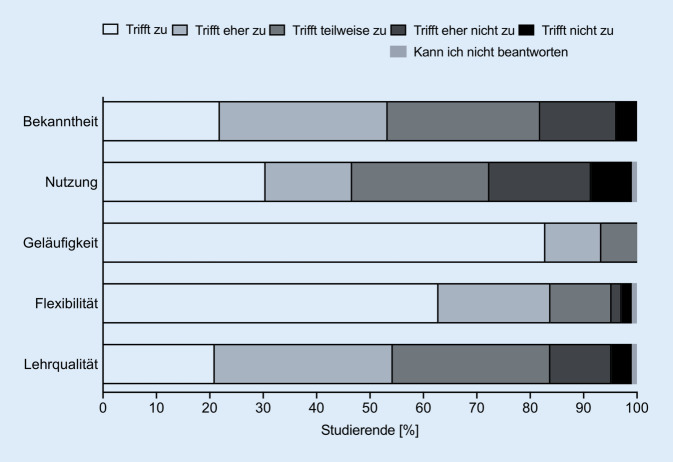

**Figure 2:**
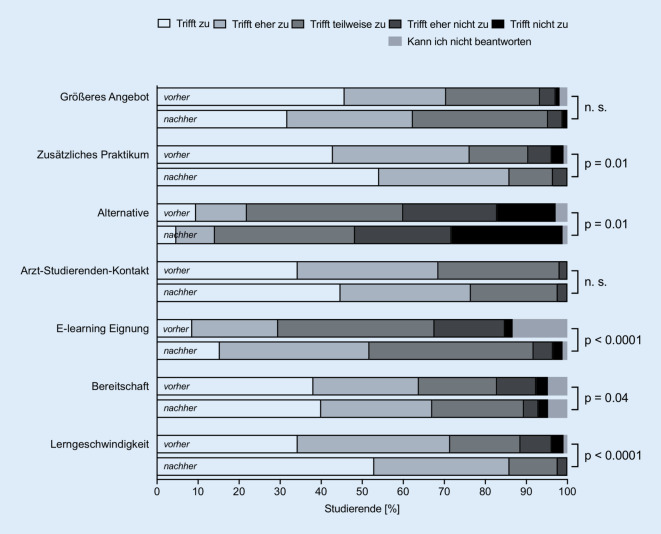

**Figure 3:**
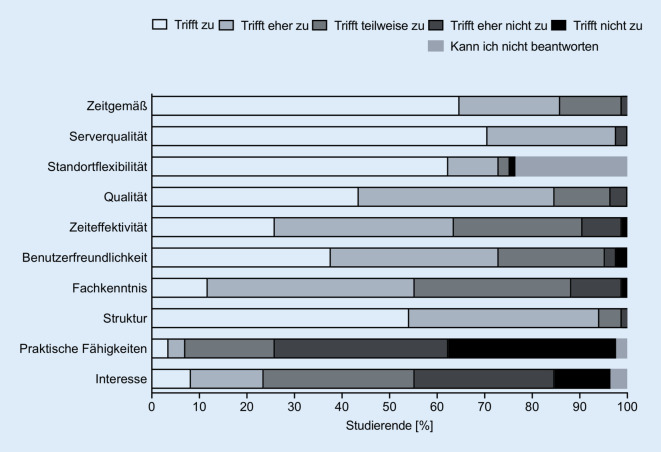

Insgesamt nahmen 105 Studierende am elektronischen Lernprogramm im Sommersemester 2020 teil. Alle 105 Studierenden füllten den ersten Evaluationsbogen aus (Abb. 1) und nahmen an den 4 Multiple-Choice-Tests teil. Den zweiten Evaluationsbogen füllten 85 der 105 Studierenden aus (Abb. 3). Von 84 Studierenden lagen Daten von beiden Zeitpunkten (vor und nach Absolvierung der elektronischen Lernkurse) vor und konnten zum statistischen Vergleich der Fragen 1 bis 7 herangezogen werden (Abb. 2). Die Fragen der Evaluationsbögen sind Tab. 1 zu entnehmen. Die deskriptiven Angaben zu den Antworten auf die jeweiligen Fragebögen sind Tab. 2 und 3 zu entnehmen.

**Table 2:** 

Aussage	1	2	3	4	5	6	7	8	9	10	11	12	13
Studierende (*n*)	105	105	105	105	105	105	105	105	105	105	105	105	105
Minimum	1,0	1,0	1,0	1,0	1,0	1,0	1,0	1,0	1,0	1,0	1,0	1,0	2,0
25%-Perzentile	1,0	1,0	2,0	1,0	3,0	1,0	2,0	1,0	1,0	1,0	1,0	2,0	3,0
Median	2,0	2,0	3,0	2,0	3,0	2,0	2,0	1,0	2,0	1,0	3,0	2,0	3,0
75%-Perzentile	3,0	3,0	4,0	3,0	4,0	2,0	3,0	2,0	3,0	1,0	4,0	3,0	4,0
Maximum	6,0	6,0	6,0	4,0	6,0	6,0	6,0	6,0	6,0	3,0	6,0	5,0	6,0
Spannweite	5,0	5,0	5,0	3,0	5,0	5,0	5,0	5,0	5,0	2,0	5,0	4,0	4,0
Mittelwert	2,1	2,3	3,2	2	3,3	2	2,5	1,6	1,9	1,2	2,6	2,5	3,3
SD	1,1	1,4	1,4	0,8	1,2	1,1	1,1	1,0	1,1	0,6	1,3	1,1	1,1

**Table 3:** 

Aussage	1	2	3	4	5	6	7	8	9	10	11	12	13	14	15	16	17	18
Studierende (*n*)	85	85	85	85	85	85	85	85	85	85	85	85	85	85	85	85	85	85
Minimum	1,0	1,0	1,0	1,0	1,0	1,0	1,0	1,0	1,0	1,0	1,0	1,0	1,0	1,0	1,0	1,0	1,0	1,0
25%-Perzentile	1,0	1,0	2,0	1,0	3,0	1,0	1,0	3,0	3,0	1,0	2,0	1,0	1,0	1,0	1,0	1,0	1,0	2,0
Median	1,0	2,0	2,0	2,0	4,0	1,0	2,0	3,0	4,0	1,0	2,0	2,0	2,0	2,0	1,0	1,0	1,0	3,0
75%-Perzentile	2,0	3,0	3,0	2,0	5,0	2,0	3,0	4,0	5,0	2,0	3,0	3,0	3,0	2,0	4,0	2,0	2,0	4,0
Maximum	4,0	6,0	6,0	4,0	6,0	4,0	5,0	6,0	6,0	4,0	5,0	5,0	5,0	4,0	6,0	4,0	4,0	5,0
Spannweite	3,0	5,0	5,0	3,0	5,0	3,0	4,0	5,0	5,0	3,0	4,0	4,0	4,0	3,0	5,0	3,0	3,0	4,0
Mittelwert	1,6	2,2	2,5	1,8	3,6	1,6	2,1	3,3	4,0	1,5	2,5	2,0	2,2	1,8	2,4	1,3	1,5	2,9
SD	0,8	1,3	1,0	0,8	1,2	0,8	0,9	1,2	1,0	0,6	0,9	1,0	1,0	0,8	2,1	0,6	0,7	0,8

**Figure 4:**
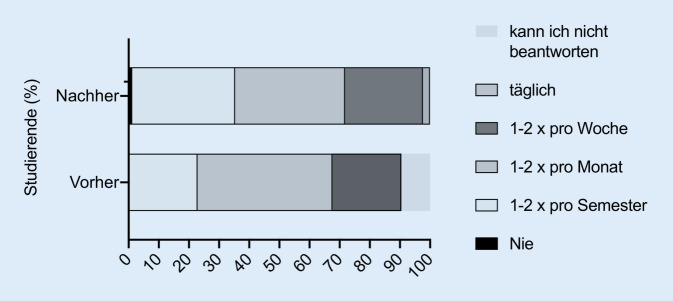

Die Antworten der Studierenden auf die Aussagen 8 bis 12 vor Durchführung der E‑Learning-Kurse sind in Abb. 1 dargestellt. Der Vergleich für die Aussagen 1 bis 7 vor und nach Durchführung des elektronischen Lernens sowie die Antworten der Studierenden auf die Aussagen 8 bis 17 nach Durchführung des elektronischen Lernens sind in Abb. 2 und 3 dargestellt. Die Angaben zur Nutzungshäufigkeit (Frage 13 bzw. 18) sind in Abb. 4 dargestellt. Im Vergleich zeigten sich signifikante Unterschiede bei den Aussagen „E-Learning in der HNO-Heilkunde sollte zusätzlich zum Anwesenheitspraktikum zur Verfügung gestellt werden.“ (*p* < 0,01), „E-Learning bietet eine gute Alternative zum herkömmlichen HNO-Anwesenheitspraktikum.“ (*p* = 0,01), „Das Fach HNO-Heilkunde eignet sich gut für den Einsatz von E‑Learning.“ (*p* < 0,0001), „E-Learning würde zu einer höheren Bereitschaft der Studierenden zur Teilnahme an Kursen und Vorlesungen führen.“ (*p* = 0,04) und „E-Learning empfinde ich als hilfreich, da es meine eigene Lerngeschwindigkeit berücksichtigt.“ (*p* < 0,0001).

Zur Lernerfolgskontrolle wurde nach jedem Lernkurs ein Multiple-Choice-Test durchgeführt. Die Gesamtbestehensrate der Studierenden lag bei allen 4 Lernkursen (äußerer Hals [I], Rachen/Kehlkopf [II], Nase [III], Ohr [IV]) bei 100 %. Das durchschnittliche prozentuale Ergebnis lag für [I] bei 91,5 %, [II] bei 95,5 %, [III] bei 89,4 %, [IV] bei 91,8 %. Das durchschnittliche prozentuale Ergebnis aller Studierenden der 4 Multiple-Choice-Tests lag bei 92 % richtig beantworteter Fragen.

## Diskussion

Aufgrund der digitalen Transformation der Lehre erscheint der Präsenzunterricht als traditionelle Lehrmethode allein nicht mehr zeitgemäß [1, 4, 8, 12]. Die Digitalisierung wird bereits seit mehreren Jahren propagiert und politisch gefördert [13]. Während im klinischen Alltag die digitale Transformation bereits stattfindet und integriert ist, erfolgte die Umsetzung in der Lehre bisher nur partiell. Angetrieben durch die aufgrund der anhaltenden COVID-19-Pandemie eingesetzten Präsenzbeschränkungen wurde innerhalb der letzten Monate ein digitales Lehrangebote für die Studierenden im Fachbereich der Hals-Nasen-Ohren-Heilkunde geschaffen [4, 14]. Die COVID-19-Pandemie hat die Entwicklung von der analogen zur digitalen Lehre folglich zwar nicht begründet, aber beschleunigt. Digitale Lehrformate haben in den letzten Jahren insbesondere im Hinblick auf international einheitliche Standards zusätzlich an Bedeutung gewonnen, und eine Überlegenheit gegenüber traditioneller Lehrmethoden wird diskutiert [15, 16]. Moderne Konzepte, die die Digitalisierung in Ausbildung von Studierenden in der Hals-Nasen-Ohren-Heilkunde implementieren, zeigen insgesamt ein hohes Interesse und eine positive Akzeptanz des Themas [17]. Dennoch erfordert das Format des elektronischen Lernens eine gute Konzeptualisierung, um einen kritischen Informationsgehalt nicht zu überschreiten [23]. In der Literatur wird eine Gleichwertigkeit bezüglich der Effektivität von traditionellem Präsenzunterricht und modernen digitalen Formaten beschrieben [17–19].

Die Wissensinhalte für das Fachgebiet der Hals-Nasen-Ohren-Heilkunde sind aufgrund der exzellenten Visualisierungsmöglichkeiten besonders geeignet. Web-Content-Management-Systeme (elektronische Inhaltsverwaltungssysteme) stellen besonders für diese Inhalte sehr gut geeignete Lernplattformen dar. ILIAS, als kostenloses, von vielen Hochschulen eingesetztes System, bietet die Möglichkeit, den Kursinhalt standardisiert und automatisiert den Studierenden zur Verfügung zu stellen und sorgt für die Vereinheitlichung und Sicherstellung der Lehrqualität bei verschiedenen Dozenten und unterschiedlichem didaktischen Vorgehen. Dozenten können die Inhalte nicht nur einfach und schnell überarbeiten, sondern auch den zu vermittelnden Inhalt anpassen und ansprechend sowie hochwertig präsentieren [4]. Letzteres ist besonders für Fächer wie die Hals-Nasen-Ohren-Heilkunde, welche im Vergleich zu den großen klinischen Fächern im Vorlesungsplan geringer repräsentiert sind, wichtig [4, 20, 21].

Die Studierenden haben die Kontrolle über den Inhalt, die Lernsequenz, das Lerntempo und die Fraktionierung der Lerninhalte, wodurch sie ihre Erfahrungen so anpassen können, dass sie die persönlichen Lernziele erfüllen können. Das elektronische Lernen ist im Sinne der Effizienz als vorteilhaft einzustufen [4, 22]. Die automatisierte Verfolgung und Berichterstattung der Aktivitäten der Studierenden verringert den Verwaltungsaufwand der Fakultät. Darüber hinaus kann das elektronische Lernen so gestaltet werden, dass es eine Ergebnisbewertung umfasst, um festzustellen, ob ein Lernerfolg stattgefunden hat. Die Studierenden sehen und wünschen sich elektronische Lehre nicht als Ersatz für die traditionelle Präsenzlehre, sondern insbesondere als Ergänzung dazu [4, 23]. Diese Kombination ist Teil einer „Blended-Learning-Strategie“ [22]. „Blended learning“ oder „integriertes Lernen“ bezeichnet eine Lernform, die eine didaktisch sinnvolle Verknüpfung von traditionellen Präsenzveranstaltungen und modernen Formen von elektronischem Lernen anstrebt. Das Konzept verbindet die Effektivität und Flexibilität von elektronischen Lernformen mit den sozialen Aspekten der „Face-to-Face-Kommunikation“ sowie dem praktischen Erlernen von Tätigkeiten. Innovationen bei Technologien für die elektronische Lehre deuten auf eine Revolution in der Bildung hin, die es ermöglicht, das Lernen zu individualisieren (adaptives Lernen), die Interaktion der Studierenden mit anderen zu verbessern (kollaboratives Lernen) und die Rolle des Dozenten zu verändern [22].

Konventionelle Wissensaneignung mit Lehrbuch und bibliothekarischem Lernen ist abhängig von der Selbstmotivation und der Fähigkeit zu lernen sowie finanziell herausfordernd. Obwohl das elektronische Lernen den Studierenden zeitliche und örtliche Ungebundenheit bietet und wesentliche der o. g. Nachteile abmildert, fühlen sich die Studierenden häufig isoliert [4]. Besonders der persönliche Austausch, welcher insbesondere beim Lernen gegenseitige Diskussionen anzuregen vermag, fehlt.

Nur im Praktikum mit tatsächlicher Präsenz können Patientengespräche und Untersuchungen selbstständig durchgeführt werden. Elektronisches Lernen eignet sich nicht, um die kommunikative Kompetenz in der Arzt-Patienten-Beziehung, speziell aber auch zwischenmenschliche Fähigkeiten zu vermitteln bzw. diese zu verbessern [23]. Einblicke in den echten Klinikalltag sind für die Entwicklung der Studierenden unerlässlich.

Zusammenfassend lässt sich sagen, dass sich vonseiten der Studierenden eine hohe Akzeptanz und Bereitschaft zur Teilnahme an Kursen und am elektronischen Lernen zeigt. Eine durchweg positive Beurteilung der Lerngeschwindigkeit und Fachkenntnis kann festgestellt werden.

In dieser Studie wurde ein Modell zur Konzeptualisierung durch die Erzeugung eines innovativen elektronischen Lernprogramms mit hochauflösendem Videomaterial vorgestellt. Insgesamt wurde das Programm von den Studierenden positiv aufgefasst und regelmäßig genutzt. Die Evaluation lässt darauf schließen, dass durch die Nutzung von digitalen Ressourcen und neuen Medien eine moderne und innovative Methode zur Erweiterung der Lehre und Verfügbarkeit von Informationsvermittlung entsteht [18]. Entwicklungen im Bereich des elektronischen Lernens bilden zukünftig die Grundlage für eine Revolutionierung im Bildungssystem [22]. Elektronisches Lernen steht somit nicht in Konkurrenz zur Präsenzlehre in den Einrichtungen der Krankenversorgung, sondern ersetzt vielmehr konventionelle Lehrbücher und die Wissensvermittlung außerhalb der Universitätsklinik.

## Limitationen

Diese Studie ist limitiert durch den fehlenden Vergleich zu einer Kontrollgruppe. In den vergangenen Semestern wurde keine Zwischenabfrage des erlernten Wissens vorgenommen, welche einen unmittelbaren Vergleich des im elektronischen Lernprogramm generierten Wissens zum bisherigen Anwesenheitspraktikum zulässt. Durch die Einschränkungen der Präsenzlehre im Sommersemester 2020 konnte die Bewertung der elektronischen Lernmodule und Beantwortung der Multiple-Choice-Tests am Ende des Semesters lediglich an den Studierenden überprüft werden, welche ausschließlich das elektronische Lernprogramm absolvierten. Um die Effektivität des elektronischen Lernens gegenüber dem konventionellen Anwesenheitspraktikum zu untersuchen, ist eine weitere prospektive Evaluation, inklusive Vergleich zu den Studierenden, welche das konventionelle Praktikum mit und ohne elektronisches Lernprogramm absolvieren, vorgesehen. Außerdem erfolgte neben dem Ausbau des elektronischen Lernprogramms der Klinik und Poliklinik für Hals-Nasen-Ohren-Heilkunde auch in anderen Kliniken der Universitätsmedizin die Erweiterung von verschiedenen Lernprogrammen. Aus diesem Grund wurden einige Aussagen der Evaluationsbögen bewusst offen formuliert, um die allgemeine Kenntnis und bisherige Auseinandersetzung mit dem Thema abzufragen. Es ist deshalb davon auszugehen, dass die Antworten der Studierenden nicht allein auf das Angebot der Klinik für Hals-Nasen-Ohren-Heilkunde fokussiert waren, sondern auch durch das insgesamt gewachsene Angebot an digitalen Lehrmethoden mit beeinflussten wurden. Nichtsdestotrotz wurde neben der insgesamt positiven Beurteilung durch die fachspezifisch formulierten Multiple-Choice-Fragen auch der Inhalt der Kurse abgefragt und eine gute Fachkenntnis der Hals-Nasen-Ohren-Heilkunde festgestellt.

## Fazit für die Praxis

- ILIAS bietet die Möglichkeit, Texte, Bilddateien und Videos dem Studierenden in einem definierten Lernkontext zur Verfügung zu stellen.
- Der Studierende kann das erworbene Wissen durch Multiple-Choice-Tests selbstständig überprüfen und bekommt direkt ein Feedback zur eigenen Leistung.
- Das elektronische Lernen kann das herkömmliche Blockpraktikum für Hals-Nasen-Ohren-Heilkunde nicht vollständig ersetzen, bietet aber eine gute Alternative bei Ausgangsbeschränkungen im Rahmen von Erkrankungen oder Pandemien (COVID-19-Pandemie).
- Als zusätzliches Angebot zum Anwesenheitspraktikum wird elektronisches Lernen von den Studierenden stark geschätzt.
- Die Resultate lassen auf eine große Akzeptanz bei den Studierenden sowie auf eine standort- und zeitunabhängige Verwendung schließen.
- Elektronisches Lernen steht nicht in Konkurrenz, sondern ist komplementär zur Präsenzlehre in den Einrichtungen der Krankenversorgung.
